# Daming capsule restores endothelial dysfunction induced by high-fat diet

**DOI:** 10.1186/1472-6882-12-21

**Published:** 2012-03-24

**Authors:** Rong Zhang, Huifang Niu, Ning Wang, Lihua Sun, Yi Xu, Ruibo Zhao, Xiang Ban, Yao Yu, Baofeng Yang, Jing Ai

**Affiliations:** 1 Department of Pharmacology, (the State-Province Key Laboratory of Biomedicine and Pharmaceutics), and Key Laboratory of Cardiovascular Research, Ministry of Education, Harbin 150086, Peoples' Republic of China; 2 Department of Pathology, Harbin Medical University, Harbin 150081, Peoples' Republic of China

## Abstract

**Background:**

Daming capsule (DMC), a traditional Chinese formula, has a lipid-modulating action with reduced adverse side effects as compared with other lipid lowering compounds. Since endothelial dysfunction often accompanies the hyperlipidemic state, we hypothesize that DMC might restore endothelial dysfunction produced by a high-fat (HF) diet. Importantly, we also investigate possible mechanisms involved in mediating the effects of DMC on vascular reactivity.

**Methods:**

Rats were divided into four groups: control, HF diet, HF mixed DMC diet, HF mixed atorvastatin (ATV) diet. After 30 days, the thoracic cavity was exposed to remove the thoracic aorta for (i) histological examination; (ii) measurement of endothelial nitric oxide synthase (eNOS) by western blot; and (iii) tension study of thoracic aortic ring.

**Results:**

HF diet induced significant attenuation in the contraction and relaxation of rat aortic rings. Treatment with DMC significantly improved the relaxation of the aortic rings as compared with those from HF rats (*P *< 0.05), which was abolished by a nonspecific NOS inhibitor L-NAME. Moreover DMC significantly restored the decrease in eNOS expression induced by HF diet. Similar results were found in histopathologic changes. DMC failed to restore the loss of vasocontraction of aorta explained by an impairment of ATP-sensitive K^+ ^channels (K_ATP_) on the structure and/or function. DMC exerted the same protective effect as ATV, a positive control drug, on vascular injury produced by HF diet.

**Conclusion:**

DMC partially protects the aorta from HF-induced endothelial dysfunction via upregulation of the expression of eNOS.

## Background

Hyperlipidemia is an important independent risk factor for cardiovascular diseases. Hyperlipidemia is accompanied by vascular disease such as: atherosclerosis, angiostenosis and blocking, which may induce hypertension, cerebral apoplexy, myocardial infarction, and even sudden cardiac death. Accumulating evidence indicates that a high-fat diet induces both systemic and tissue oxidative stress and the development of early vascular lesions [1]. Impaired endothelial function, an early hallmark of atherogenesis, was observed in rats and healthy volunteers fed high-saturated fat and high-sucrose meals [2,3]. Moreover, nitric oxide (NO) produced by endothelium was inhibited by high-fat diet [4]. Hyperlipidemia and oxidation of low density lipoprotein (LDL) induce vascular smooth muscle cell growth [5] and hyperlipidemia may alter the vascular response to vasodilators [6]. The regulation of vascular tone is important for maintaining adequate perfusion to critical organs and is regulated by factors released from the endothelium and by other components such as receptors, ion channels and signaling pathways [7,8]. Therefore, it's valuable to investigate the mechanisms of various compounds on controlling vessel tone in vessel-related diseases.

Compared with Western medicine, the traditional Chinese medicine formula has a prominent advantage due to a stable curative effect with reduced toxicity. It simultaneously targets multiple physiological processes to arouse the whole body's potentiality to recover to health. The traditional Chinese medicine compatibility emphasizes *jun, chen, zuo, shi *(monarch, minister, adjuvant and messenger) with proper herbs and relevant dosage to synergize the desirable effects and minimize side effects integrally [9]. Daming capsule (DMC) was designed and carefully formulated in accordance with the rule of the traditional Chinese medicine theory comprising: *Rheum Palmatu, Cassia obtusifolia L, Salvia miltiorrhiza *and *Panax ginseng C.A*.. *Rheum Palmatu *is regarded as monarch component for its actions on promoting digestion to remove food retention and improving blood circulation to dissipate blood stasis (Pharmacopoeia of the People's Republic of China, 2010). *Cassia obtusifolia L, Salvia miltiorrhiza *and *Panax ginseng C.A*. are considered as minister or adjuvant components to enhance the pharmacological functions and compensate for the side effects of *Rheum Palmatu *[9]. Our previous clinical study reported that DMC had a lipid-lowering action with lower adverse side effects and significantly decreased serum total cholesterol and LDL cholesterol indicating that it might be a good candidate for the treatment of hyperlipidemia [10]. Further study demonstrated that DMC could reverse the prolonged QT and PR interval and improve heart function [9] and also restored impaired baroreflexes in STZ-induced diabetic rats with hyperlipidemia [11]. There is no report related to the role of DMC on the regulation of vascular morphology or function that is negatively impacted by hyperlipidemia. This study was designed to investigate whether DMC has effects on vascular reactivity and to test the hypothesis that DMC may affect endothelial nitric oxide synthase (eNOS) in endothelium and in turn contribute to the restoration of endothelial dysfunction of aorta from rats fed a high-fat (HF) diet.

## Methods

### Chemicals and herbal materials

The formula of DMC was designed by Professor Baofeng Yang in the department of Pharmacology of Harbin Medical University (Patent No.: ZL03109063.X) and produced by Harbin Yida Ltd as reported in our previous publications [9-11]. Briefly, the plants were harvested from corresponding provinces and collected at appropriate season. The grounded powders were capsulated at a ratio of 12:12:6:1 of *Rheum Palmatu, Cassia obtusifolia L, Salvia miltiorrhiza *and *Panax ginseng C.A.*. The entire process was supervised according to the policy of the State Food and Drug Administration of P.R.China. Quality control was performed by marker compound chrysophanol using HPLC analysis to quantify total anthraquinones in DMC. The content of chrysophanol was more than 1.5 mg in each capsule (300 mg) estimated from standard calibration curve. Atorvastatin (ATV) was purchased from Huirui Pharmaceutical Co., Ltd., China. All reagents used in our vascular reactivity studies were purchased from Sigma, Saint Louis, MO, USA.

### Animals

Male Wistar rats (200-230 g, *n *= 40) were obtained from the Animal Center of the 2nd Affiliated Hospital of Harbin Medical University, China, and housed in a room with controlled temperature of 23 ± 1°C and humidity of 55 ± 5% under a 12 h-12 h light-dark cycle. All experimental procedures and protocols used in this investigation received approval by the ethic committees of Harbin Medical University.

### Establishment of HF model

The HF emulsion was prepared as previously described [9-11]. Fat emulsion comprised lard (20%), thyreostat (1%), cholesterol (5%), sucrose (5%), saccharu (5%) and sodium glutamate (1%) in 20% Tween 80 and 30% (v/v) propylene glycol. The diet was kept at 4°C before use. Rats were randomly divided into four groups (*n *= 10 in each group) as follows: control group, HF group, HF + DMC (100 mg/kg/d, the optimal dose from previous study [9]) group and HF + ATV (7.2 mg/kg/d, dissolved with 0.5% carboxymethylcellulose sodium) group. The control group received 0.9% NaCl (10 ml/kg/d) and the HF rats received the HF emulsion (10 ml/kg/d) by intragastric administration for 30 days. DMC and ATV was mixed in HF emulsion and administered to the rats in DMC treated group and ATV treated group, respectively.

### Vascular reactivity studies and experimental protocols

These studies were performed as previously described [12]. Briefly, the thoracic cavity was exposed to remove the thoracic aorta in anesthetized rats. Adherent fat and connective tissue was cleaned from aortas which were used for: (i) histological examination; (ii) measurement of eNOS by western blot; and (iii) tension study of thoracic aortic ring. Aortas were cut into rings (2-3 mm), mounted on a force transducer placed in an organ bath having pH-adjusted, oxygenated Ringer's solution (mM: NaCl 118, KCl 4.7, MgSO_4 _0.6, KH_2_PO_4 _1.18, CaCl_2 _2.5, glucose 10 and NaHCO_3 _27, pH 7.4) at 37°C. Rings were initially loaded with 1.5 g tension (basal tension) by incremental application over 30 min and then equilibrated for an additional 30-40 min before the studies were started. A dose-dependent constriction to phenylephrine (PE) (10^-6 ^M to 10^-5 ^M) followed by measurement of vasorelaxation in response to acetylcholine (ACh) (10^-5 ^M to 10^-4 ^M) was examined. Aortic rings were washed repeatedly with Ringer's solution until tone recovered to basal level and then treated with different inhibitors according to protocols as follows. Reactivity of the rings was again measured to 10^-5 ^M PE and 10^-4 ^M ACh. Vasorelaxation to ACh was calculated as (tension PE - tension ACh)/tension PE (the percent change in reactivity to 10^-4 ^M ACh after10^-5 ^M PE). At termination of the experiment, KCl (60 mM) was applied to detect the non-receptor mediated response of the rings. Tension data were relayed from the pressure transducers to a signal amplifier. BL-420E + signal analysis software (Chengdu, China) was employed to describe the isometric changes in force of the aortic rings.

*Protocol 1: *To determine the effect of K^+ ^channels, 4-amionpyridine (4-AP, 3 mM, K_V _channel blocker), tetraethylammonium (TEA, 1 mM, BK_Ca _channel blocker) or glyburide (GLYB, 1 μM, K_ATP _channel blocker) were pre-incubated for 30 min respectively before adding PE and ACh.

*Protocol 2: *To determine the effect of eNOS, N^G^-Nitro-L-arginine Methyl Ester (L-NAME, 10^-4 ^M, a nonspecific NOS inhibitor) was pre-incubated for 30 min before adding PE and ACh.

### Histology

The thoracic aorta segments were taken and fixed in zinc formalin for 24 to 48 h then processed using a Sakura Tissue Tek VIP5 processor. Processing was performed using 70%, 80%, 95% and 100% ethanols, xylene and paraffin. Following processing, samples were oriented (embedded) in paraffin and sectioned at 4 μm using a microtome. Sections were stained with hematoxylin and eosin (H&E) for histological examination.

### Western blot analysis

The thoracic aorta segments were homogenized in lysis buffer (RIPA buffer 60%, SDS 40% and protease inhibitor cocktail 1%) on ice, then centrifuged at 14,000 rpm at 4°C for 30 min to remove the insoluble pellet. Protein concentration in the supernatant was determined by the Biorad DC Protein Assay (Biorad, Hercules, CA, USA). Equal amounts of protein (60 μg) were loaded on 8% SDS-PAGE gel. The lysates were resolved by electrophoresis (100 V for 1 h) and transferred onto nitrocellulose membranes. After being blocked in 5% nonfat milk for 2 h, the membranes were treated with primary antibody for rabbit anti-eNOS (sc-654, Santa cruz, CA, USA), overnight at 4°C and washed 5 times before incubating with secondary antibody (1:2000) for 1 h. Blots were detected with the Odyssey infrared imaging system (Licor, USA). Protein loading was confirmed using GAPDH (1:2000, Sigma, Saint Louis, MO, USA) as an internal control.

### Statistical analysis

Data were calculated as means ± S.E.M (standard error of the means). The changes in vasoreactivity were expressed as the percent increase in tone from the baseline. The differences in mean values among the experimental groups were measured using two-tailed analyses of variance (ANOVA) followed by Dunnetts's test. Differences were considered statistically significant at *P *< 0.05.

## Results

### The dose-response reactivity curves to PE and ACh in aortic rings

**Figure 1:**
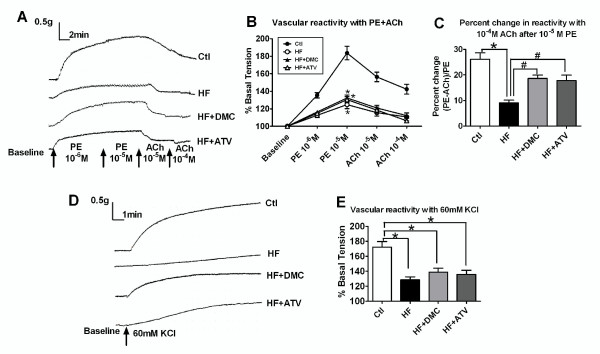
**High-fat-induced loss of vascular reactivity of aortic rings and mitigation by DMC and ATV**. (**A**) Typical tracing and (**B**) Representative line graph showing increased contraction and relaxation by increasing concentration of PE and ACh. (**C**) The percent change in relaxation ((tension PE - tension Ach)/tension PE) to 10^-4 ^M ACh after10^-5 ^M PE. (**D**) Typical tracing and (**E**) Vascular tension to 60 mM KCl in all groups. Data presented as mean ± S.E.M. and expressed as percentage of baseline tension. * *P *< 0.01 vs. control group, # *P *< 0.05 vs. HF, *n *= 16-18 rings from 6-7 rats.

In vascular reactivity studies increasing concentrations of PE and ACh were applied to detect vascular contraction and relaxation, respectively. Aortic rings exhibited dose-dependent contraction and relaxation to PE (10^-6 ^M to 10^-5 ^M) and ACh (10^-5 ^M to 10^-4 ^M), respectively (Figure 1A, typical tracing of vascular reactivity), indicating that 10^-5 ^M PE and 10^-4 ^M ACh were maximal and therefore these concentrations were used in the remaining experiments. As shown in Figure 1B and 1C, the vascular contraction and relaxation were significantly attenuated in rats fed a HF diet (124.76 ± 4.6 vs. 183.66 ± 7.72 in contraction, *P *< 0.01; 9.09 ± 1.03 vs. 26.14 ± 2.47 in relaxation, *P *< 0.01). Treatment with DMC did not improve the loss of vasocontraction (132.3 ± 3.48) but significant mitigation was seen in the relaxation of the aortic rings from DMC treated group (18.59 ± 1.31 vs. 9.09 ± 1.03, *P *< 0.01). Same result was obtained from ATV treated rats (130.45 ± 3.42 in contraction, 17.74 ± 2.2 in relaxation). Figure 1D showed the typical tracing of vascular reactivity with KCl. A similar trend of vasocontraction was observed if those rings exposed to 60 mM KCl (Figure 1E).

### The influence of K^+ ^channels on the contraction of aortic rings

**Figure 2:**
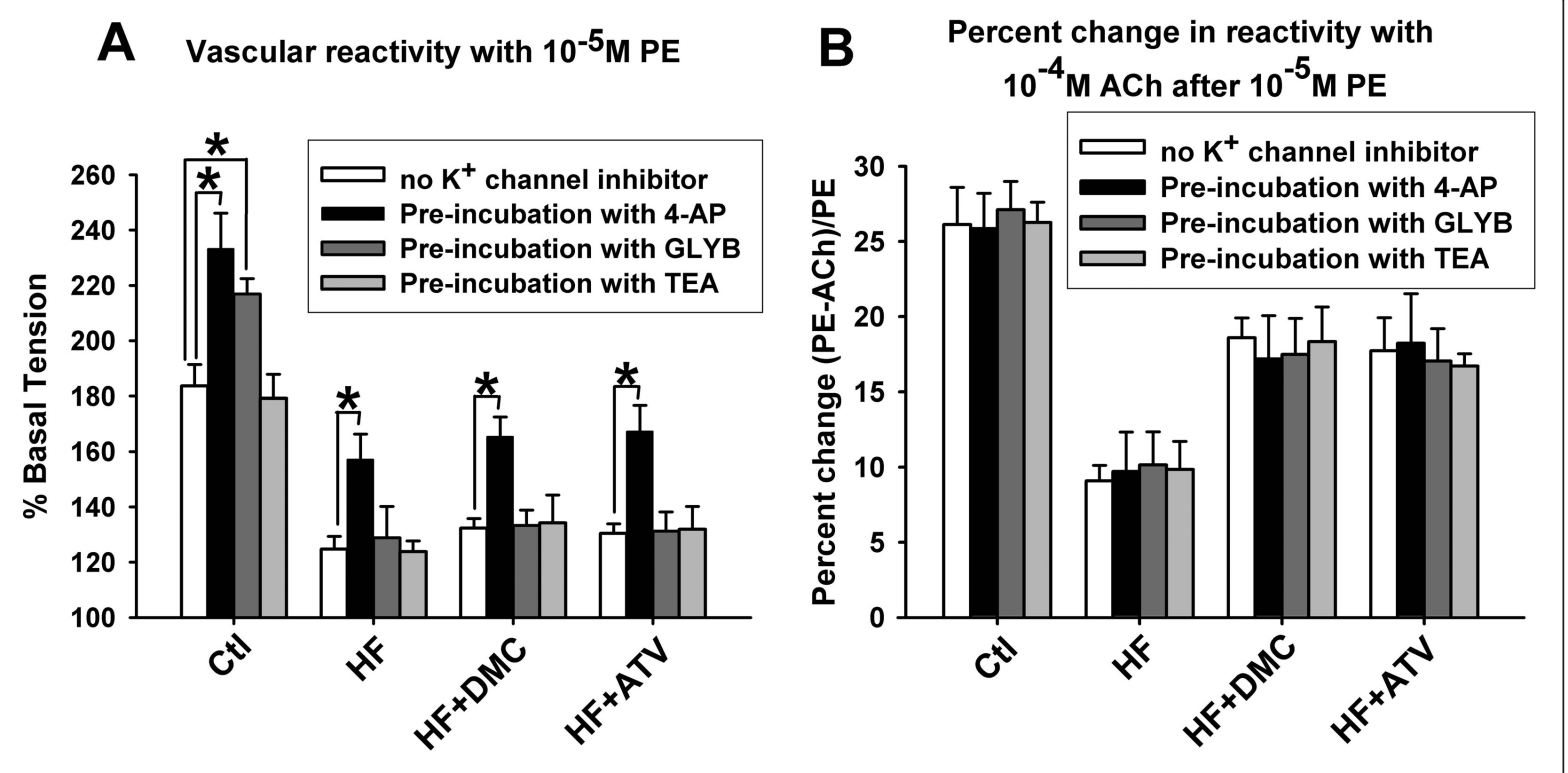
**Effect of K+ channels on the contraction of aortic rings**. (**A**) The contraction of aortic rings with 10^-5 ^M PE before and after pre-incubation with 4-AP, 3 mM, K_V _channel blocker; TEA 1 mM, BK_Ca _channel blocker and GLYB, 1 μM, K_ATP _channel blocker respectively for 30 min. (**B**) The percent change in relaxation ((tension PE - tension Ach)/tension PE) to 10^-4 ^M ACh after 10^-5 ^M PE before and after pre-incubation with three K^+ ^channel blockers respectively. Data presented as mean ± S.E.M. and expressed as percentage of baseline tension. * *P *< 0.01 vs. contraction value of corresponding group before pre-incubation of K^+ ^channel blocker, *n *= 8-10 rings from 5-6 rats.

As shown in Figure 2A, pre-incubation of 4-AP, a K_V _channel blocker, significantly increased contraction of aortic rings in control rats, indicating that K_V _channels were involved in the process of constriction. Similar results were observed in HF, DMC and ATV treated rats, suggesting K_V _channels play the same role in all groups. Pre-incubation of GLYB, a K_ATP _channel blocker, significantly enhanced the vasocontraction in control rats but not in the other groups. These results demonstrate that blockade of K_ATP _channels affected vasoconstriction of rats fed a HF diet. The structure and/or function of K_ATP _channels may thus be altered in HF model and were not improved by the treatment of DMC and ATV. There was no change of vasocontraction in all groups after pre-incubation of TEA, BK_Ca _channel blocker, indicating BK_Ca _channels were not involved in the contraction of aortic rings. The relaxation of aortic rings of four groups weren't changed after pre-incubation of K^+ ^channel blockers (Figure 2B).

### The effect of eNOS on the relaxation of aortic rings

**Figure 3:**
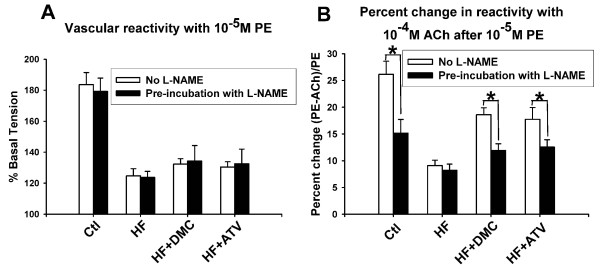
**Effect of eNOS on the relaxation of aortic rings**. (**A**) The contraction of aortic rings with 10^-5 ^M PE before and after pre-incubation of L-NAME, 10^-4 ^M, eNOS inhibitor. (**B**) The percent change in relaxation (tension PE - tension ACh/tension PE) to 10^-4 ^M ACh after 10^-5 ^M PE before and after pre-incubation of L-NAME. Data presented as mean ± S.E.M. and expressed as percentage of baseline tension. * *P *< 0.01 vs. relaxation value of corresponding group before pre-incubation of L-NAME, *n *= 8-10 rings from 5-6 rats.

Pre-incubation with L-NAME (a non-selective NOS inhibitor) did not alter the contraction of rings to PE significantly in all groups (Figure 3A). After applying ACh there was a significant attenuation of relaxation in control (15.15 ± 2.57 vs. 26.14 ± 2.47, *P *< 0.01), DMC treated rats (11.92 ± 1.25 vs. 18.59 ± 1.31, *P *< 0.01) and ATV treated rats (12.55 ± 1.38 vs. 17.74 ± 2.2, *P *< 0.01) but not in those fed a HF diet (8.21 ± 1.16 vs. 9.09 ± 1.03) (Figure 3B). These data supported the idea that eNOS participated in the relaxation of aortic rings in control, DMC and ATV treated rats. However, HF diet affected eNOS via a certain mechanism.

### Histological characters of aortas

**Figure 4:**
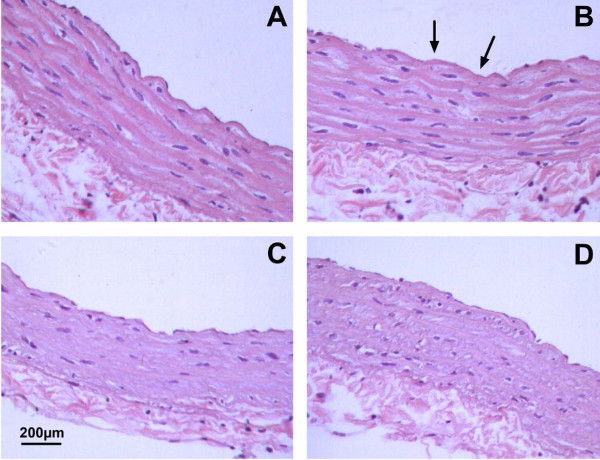
**Representative sections (4 μm thick) of H&E stained of thoracic aorta**. (**A**) Control rats. (**B**) HF rats, with considerable damage in endothelial cells including disorder arrangement, unclear borderline and partial deletion (arrow). (**C**) HF + DMC rats, with less injury than observed in HF rats. (**D**) HF + ATV rats, with less injury than observed in HF rats. Scale bars represent 200 μm.

Representative histological sections of aorta were shown in Figure 4. Figure 4A showed the normal structure of the control aorta. Considerable damage in endothelial cells was noted in HF group such as disordered arrangement, unclear borderline, and even partial deletion. There was no change in the layer of smooth muscle (Figure 4B). In DMC and ATV treated groups, these pathological injuries in endothelial cells were recovered to normal (Figure 4C, D).

### DMC restored eNOS expression in aortic endothelial cells attenuated by HF diet

**Figure 5:**
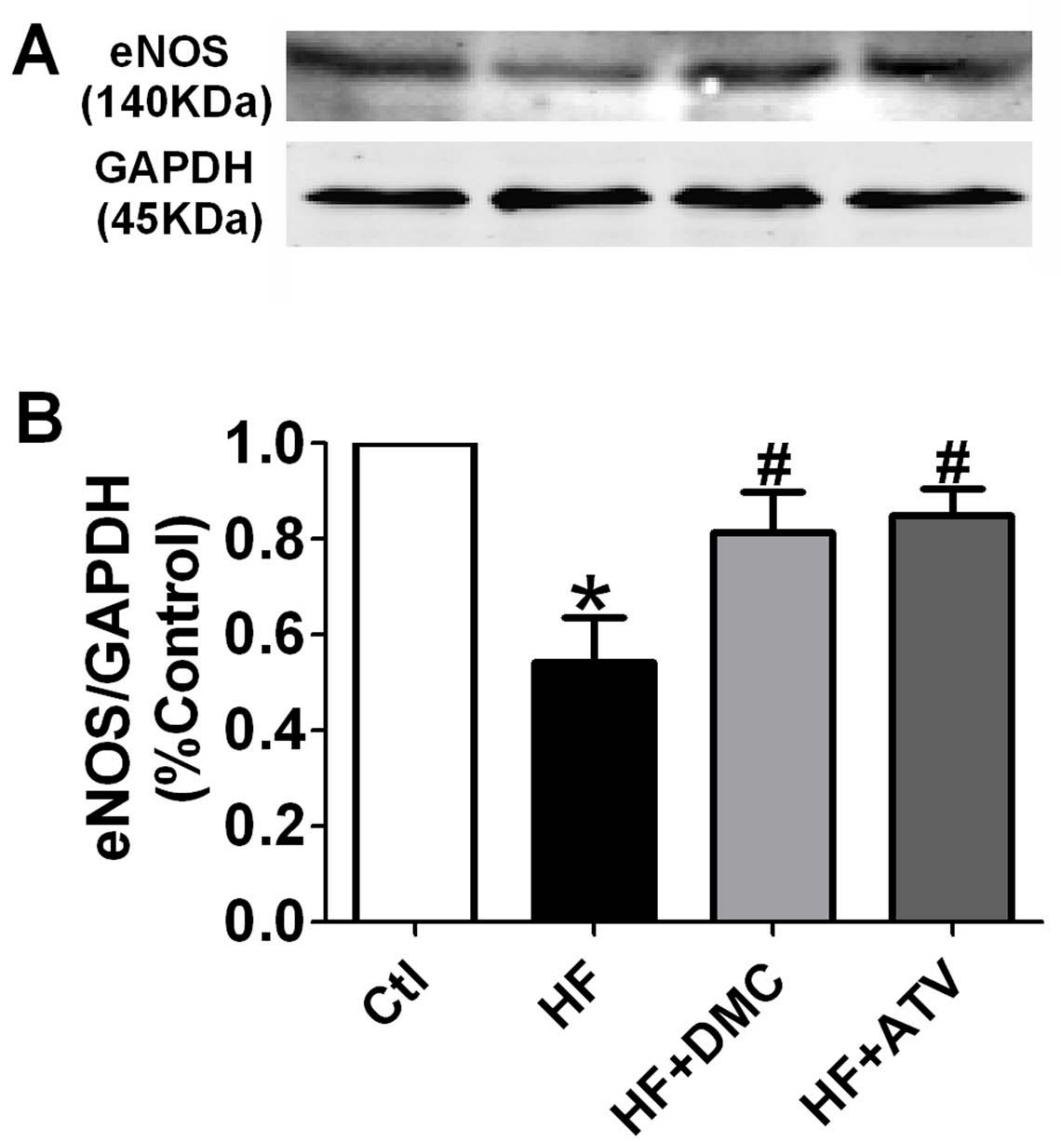
**High-fat-induced attenuation of eNOS protein expression in thoracic aorta and mitigation by DMC and ATV**. (**A**) Representative examples of western blot assay of eNOS (140 kDa) protein are shown including GADPH expression (45 kDa) as a loading control. (**B**) Statistical bar graph indicates the result of densitometric analysis of the bands as normalized to the quantity of GADPH protein. These ratios were normalized to control. * *P *< 0.01 vs. control group, # *P *< 0.05 vs. HF, *n *= 4.

eNOS expression was assayed by western blotting of the aortic homogenate. As shown in Figure 5A, HF caused the reduction of eNOS expression as observed by a weaker signal versus control band. DMC and ATV treated group showed more intense bands indicating increased eNOS expression. Protein levels of GAPDH remained unaltered demonstrating equal protein loading. A summary of these data was presented in Figure 5B.

## Discussion

The principal finding of this study was that DMC could partially protect the aorta from HF-induced endothelial dysfunction via upregulating the expression of eNOS. And we discovered that DMC failed to improve the impairment of contraction of aorta induced by HF on account of the change of K_ATP _channels on structure and/or function.

It has been recognized that vascular smooth muscle cells contribute to the contracting function while the endothelium modulates this contraction via the release of relaxing factors. In this study we applied PE and ACh to investigate the contraction and relaxation of aorta respectively after HF diet with or without DMC and ATV intervention. PE is a α_1_-adrenergic receptor agonist used extensively as a vasoconstrictor [13]. ACh is an endothelial-dependent vasodilator and a well-known method for monitoring the status of the vascular endothelium [14]. Our result demonstrated that HF diet induced significant attenuation of the vasocontraction and relaxation, indicating impaired vascular responsiveness and elasticity caused by the injury of smooth muscle layer as well as endothelium, which was consistent with those previously shown [1-3,6]. Treatment with DMC failed to mitigate the contraction of smooth muscle cells but it restored vascular endothelial dilation function. NO, as the most important endogenous vasodilator agent, is generated by the catalysis of L-arginine by eNOS which is a critical regulator of vascular tone [15]. Therefore, it is necessary to investigate the function of eNOS in aortas of different groups. Employment of a non-specific NOS inhibitor (L-NAME) indicated the participation of eNOS in vasorelaxation in DMC treated group as well as control rats. However HF diet impacted the function of eNOS via a certain mechanism therefore resulted in the abolishment of relaxation. Further evidence was obtained by the expression of eNOS in western blot analysis. Treatment with DMC recovered the decrease of eNOS expression induced by HF diet. We infer that the activity and expression of eNOS were involved in the mechanism of DMC for mitigation. Similar results were found in histopathologic examination with endothelial damage in HF rats. This pathologic damage was not found in rats with DMC treatment. Therefore we conclude that DMC protected, at least in part, the endothelial dysfunction of aorta in high-fat diet rats. Lipid-modulating drugs statins (HMG-CoA reductase inhibitors) are a milestone to lower cholesterol levels in blood and become one of the most powerful pharmacological strategies in the treatment of cardiovascular diseases [16]. Statins could relax vascular tone importantly by endothelium-dependent pathway [17]. It exerts cholesterol-independent vasoprotective effects by upregulating endothelial nitric oxide synthase and decreasing superoxide production [18]. In this study atorvastatin (ATV) was applied as a positive control to DMC. Our results were consistent with these previous reports and ATV has the same protective effect on endothelial dysfunction as DMC.

K^+ ^channels play an important role in the regulation of vascular function through inflowing or outflowing K^+ ^and Ca^2+ ^current. There are at least four different classes of K^+ ^channels, including inward rectifier K^+ ^channels (K_IR_), ATP-sensitive K^+ ^channels (K_ATP_), voltage-gated K^+ ^channels (K_V_), and large conductance Ca^2+^-activated K^+ ^channels (BK_Ca_). It was proposed that blockade of K_ATP_, K_V_, and BK_Ca_, which lead to membrane depolarization, the activation of Ca^2+ ^channels, an increased Ca^2+ ^influx, and vasoconstriction ensues [19-21]. In this study, K^+ ^channel blockers including 4-AP, GLYB, TEA for K_V_, K_ATP_, and BK_Ca _respectively were employed to determine the contribution of particular K^+ ^channels in vascular tone of aortic rings. The results revealed that BK_Ca _channel were not involved in the contraction of aortic rings and K_V _channels were involved in this process but played the same role in all groups, indicating that the mechanism of the attenuation of aortic contraction in HF and DMC and ATV treated rats was not relevant to BK_Ca _and K_V _channels. The blockade of K_ATP _channel significantly enhanced the vasocontraction of aortic rings in control rats but not in the other groups. The results demonstrated that in normal condition K_ATP _channels participated in the constriction process of aorta, but their structure and/or function were altered in HF model and these alterations were not mitigated by the treatment of DMC and ATV. Fan LH et al. [22] proved that HF diet may impair the function and expression of K_ATP _channels in vascular smooth muscle cells by patch clamp and western blotting, which provides powerful evidence for our results. So we proposed that the impairment of K_ATP _channels might be the mechanism that DMC and ATV cannot restore the dysfunction of contraction of aorta induced by HF. Though it has been reported that pravastatin has a significant beneficial effect during myocardial ischemia, which is provided by K_ATP _channels and NO [23], our results did not show the restoration of ATV on K_ATP _channels impaired by HF model.

DMC abides by *jun, chen, zuo, shi *(monarch, minister, adjuvant and messenger) and a holistic concept of traditional Chinese medicine formula and is good at treating chronic diseases due to reduced adverse side effects even for long-term application. Here we didn't investigate each component of DMC separately because it destructed the drug interactions. Despite of a compound the quality of DMC can be controlled accurately. Chrysophanol, the criteria of quality control of *Rheum Palmatu *which is the potent component of DMC, is applied to control the stability of the formula (Pharmacopoeia of the People's Republic of China, 2010). The location and season of plant acquisition and preparation process of DMC were controlled strictly for quality assurance. DMC is a research project having independent intellectual property rights and has been applied extensively in Chinese clinic as a lipid modulating drug for ten years. DMC protects heart function in streptozocin-induced diabetic rats with hyperlipidemia [9]. Our research found the protection of DMC on vascular injury induced by HF diet and provided a powerful evidence for expanding the clinical application of DMC particularly in patients with diabetes mellitus accompanied by hyperlipidemia. Moreover our study disclosed the same protective effect of DMC and ATV on vascular endothelial dysfunction produced by HF diet. Therefore, DMC displays remarkable advantages with lower adverse side effects and provides a new candidate for prevention and therapy of vascular injury induced by hyperlipdiemia.

In our study, we did not obtain the restoration of DMC on the impairment of contraction of aorta. Maybe DMC treatment for a longer period would present an effect with more intensity in vascular reactivity. Furthermore, peripheric arteries such as tail artery and mesenteric artery could present a different effect. Definitely all these need to be further investigated. More importantly, many studies indicated that inducible nitric oxide synthase (iNOS) was relevant to vascular injury [24-26] and oxidative stress induced the expression of iNOS and subsequent generation of high concentration of NO, which could interact with reactive oxygen species (ROS) causing vascular dysfunction [26-28]. The effect of DMC on ROS and iNOS in the process of vasomotion will be elucidated by our future studies.

## Conclusions

The present findings provided the first effort to establish that DMC could partially protect the endothelial dysfunction of aorta in HF diet rats via upregulating the expression of eNOS.

## Abbreviations

DMC: Daming capsule; HF: High-fat; eNOS: Endothelial nitric oxide synthase; K_ATP_: ATP-sensitive K^+ ^channels; ATV: Atorvastatin; NO: Nitric oxide; LDL: Low density lipoprotein; PE: Phenylephrine; ACh: Acetylcholine; 4-AP: 4-amionpyridine; TEA: Tetraethylammonium; GLYB: Glyburide; L-NAME: NG-Nitro-L-arginine Methyl Ester; K_IR_: Inward rectifier K^+ ^channels; K_V_: Voltage-gated K^+ ^channels; BK_Ca_: Large conductance Ca^2+^-activated K^+ ^channels; iNOS: Inducible nitric oxide synthase; ROS: Reactive oxygen species.

## Competing interests

Professor Baofeng Yang in the department of Pharmacology of Harbin Medical University holds the patent on the formula of DMC (Patent No.: ZL03109063.X) and authorized Harbin Yida Ltd to produce the formulation. There is no competing interest between them. The authors declare that they have no competing interests.

## Authors' contributions

RZ: Designing and performing the study, analyzing the data and preparing the manuscript. HN: Performing the study and analyzing the data. NW, LS and YX: Participating in the ring study. RZ, XB: Performing the histological examination. YY: Establishing the animal model. JA: Supervising the work, providing the grant, evaluating the data, correcting the manuscript and coordinating the study. BY: Supervising the work, providing the grant and coordinating the study. All authors read and approved the final manuscript.

## Pre-publication history

The pre-publication history for this paper can be accessed here:

http://www.biomedcentral.com/1472-6882/12/21/prepub

## References

[B1] Napoli C Martin-Padura I de Nigris F Giorgio M Mansueto G Somma P Condorelli M Sica G De Rosa G Pelicci P Deletion of the p66Shc longevity gene reduces systemic and tissue oxidative stress, vascular cell apoptosis, and early atherogenesis in mice fed a high-fat diet Proc Natl Acad Sci USA 2003 100 4 2112 2116 12571362 10.1073/pnas.0336359100 PMC149967

[B2] Magne J Huneau JF Tsikas D Delemasure S Rochette L Tome D Mariotti F Rapeseed protein in a high-fat mixed meal alleviates postprandial systemic and vascular oxidative stress and prevents vascular endothelial dysfunction in healthy rats J Nutr 2009 139 9 1660 1666 19587122 10.3945/jn.109.107441

[B3] Barringer TA Hatcher L Sasser HC Potential Benefits on Impairment of Endothelial Function after a High-fat Meal of 4 weeks of Flavonoid Supplementation Evid Based Complement Alternat Med 2008 10.1093/ecam/nen048 PMC3137609 18955351

[B4] Yang N Ying C Xu M Zuo X Ye X Liu L Nara Y Sun X High-fat diet up-regulates caveolin-1 expression in aorta of diet-induced obese but not in diet-resistant rats Cardiovasc Res 2007 76 1 167 174 17599814 10.1016/j.cardiores.2007.05.028

[B5] Taylor AM Li F Thimmalapura P Gerrity RG Sarembock IJ Forrest S Rutherford S McNamara CA Hyperlipemia and oxidation of LDL induce vascular smooth muscle cell growth: an effect mediated by the HLH factor Id3 J Vasc Res 2006 43 2 123 130 16340216 10.1159/000090131 PMC2929384

[B6] Li R Xu M Wang X Wang Y Lau WB Yuan Y Yi W Wei X Lopez BL Christopher TA Reduced vascular responsiveness to adiponectin in hyperlipidemic rats-mechanisms and significance J Mol Cell Cardiol 2010 49 3 508 515 20303976 10.1016/j.yjmcc.2010.03.002 PMC2904862

[B7] Brayden JE Earley S Nelson MT Reading S Transient receptor potential (TRP) channels, vascular tone and autoregulation of cerebral blood flow Clin Exp Pharmacol Physiol 2008 35 9 1116 1120 18215190 10.1111/j.1440-1681.2007.04855.x PMC4193799

[B8] Martens JR Gelband CH Ion channels in vascular smooth muscle: alterations in essential hypertension Proc Soc Exp Biol Med 1998 218 3 192 203 9648936 10.3181/00379727-218-44286

[B9] Ai J Yan X Zhao L Lu Y Liang F Cai B Li G Lu Y Yang B The protective effect of Daming capsule on heart function in streptozocin-induced diabetic rats with hyperlipidemia Biol Pharm Bull 2009 32 8 1354 1358 19652373 10.1248/bpb.32.1354

[B10] Jing A Li-Mei Z Yan-Jie L Ben-Zhi C Yong Z Bao-Feng Y A randomized, multicentre, open-label, parallel-group trial to compare the efficacy and safety profile of daming capsule in patients with hypercholesterolemia Phytother Res 2009 23 7 1039 1042 19145637 10.1002/ptr.2654

[B11] Ai J Wang LH Zhang R Qiao GF Wang N Sun LH Lu GY Sun C Yang BF Protective effect of the daming capsule on impaired baroreflexes in STZ-induced diabetic rats with hyperlipoidemia BMC Complement Altern Med 2010 10 80 21176164 10.1186/1472-6882-10-80 PMC3022895

[B12] Zhang R Ghosh SN Zhu D North PE Fish BL Morrow NV Lowry T Nanchal R Jacobs ER Moulder JE Structural and functional alterations in the rat lung following whole thoracic irradiation with moderate doses: injury and recovery Int J Radiat Biol 2008 84 6 487 497 18470747 10.1080/09553000802078396 PMC2435093

[B13] Scicluna JK Mansart A Ross JJ Reilly CS Brown NJ Brookes ZL Reduced vascular response to phenylephrine during exposure to lipopolysaccharide in vitro involves nitric oxide and endothelin 1 Shock 2008 29 3 417 421 18437715 10.1097/shk.0b013e318142c5df

[B14] Kasprzak JD Klosinska M Drozdz J Clinical aspects of assessment of endothelial function Pharmacol Rep 2006 58 Suppl 33 40 17332669

[B15] Forstermann U Nitric oxide and oxidative stress in vascular disease Pflugers Arch 2010 459 6 923 939 20306272 10.1007/s00424-010-0808-2

[B16] Endo A A historical perspective on the discovery of statins Proc Jpn Acad Ser B Phys Biol Sci 2010 86 5 484 493 10.2183/pjab.86.484 PMC3108295 20467214

[B17] Sonmez Uydes-Dogan B Topal G Takir S Ilkay Alp F Kaleli D Ozdemir O Relaxant effects of pravastatin, atorvastatin and cerivastatin on isolated rat aortic rings Life Sci 2005 76 15 1771 1786 15698855 10.1016/j.lfs.2004.11.002

[B18] Endres M Laufs U Effects of statins on endothelium and signaling mechanisms Stroke 2004 35 11 Suppl 1 2708 2711 15375300 10.1161/01.STR.0000143319.73503.38

[B19] Ko EA Han J Jung ID Park WS Physiological roles of K + channels in vascular smooth muscle cells J Smooth Muscle Res 2008 44 2 65 81 18552454 10.1540/jsmr.44.65

[B20] Borbouse L Dick GM Payne GA Berwick ZC Neeb ZP Alloosh M Bratz IN Sturek M Tune JD Metabolic syndrome reduces the contribution of K + channels to ischemic coronary vasodilation Am J Physiol Heart Circ Physiol 2010 298 4 H1182 H1189 20118408 10.1152/ajpheart.00888.2009 PMC2853415

[B21] Hodnett BL Xiang L Dearman JA Carter CB Hester RL K(ATP)-mediated vasodilation is impaired in obese Zucker rats Microcirculation 2008 15 6 485 494 19086258 10.1080/10739680801942240 PMC2788296

[B22] Fan LH Tian HY Yang ML Ma AQ Hu Z Bai XJ Cao YX High-fat diet may impair K(ATP) channels in vascular smooth muscle cells Biomed Pharmacother 2009 63 2 165 170 18339514 10.1016/j.biopha.2008.01.005

[B23] Kawabata H Ryomoto T Ishikawa K Role of cardiac ATP-sensitive K + channels induced by HMG CoA reductase inhibitor in ischemic rabbit hearts Hypertens Res 2001 24 5 573 577 11675953 10.1291/hypres.24.573

[B24] Behr D Rupin A Fabiani JN Verbeuren TJ Distribution and prevalence of inducible nitric oxide synthase in atherosclerotic vessels of long-term cholesterol-fed rabbits Atherosclerosis 1999 142 2 335 344 10030385 10.1016/s0021-9150(98)00254-8

[B25] Kibbe M Billiar T Tzeng E Inducible nitric oxide synthase and vascular injury Cardiovasc Res 1999 43 3 650 657 10690336 10.1016/s0008-6363(99)00130-3

[B26] Olukman M Orhan CE Celenk FG Ulker S Apocynin restores endothelial dysfunction in streptozotocin diabetic rats through regulation of nitric oxide synthase and NADPH oxidase expressions J Diabetes Complications 2010 24 6 415 423 20226688 10.1016/j.jdiacomp.2010.02.001

[B27] Rocha JT Hipólito UV Callera GE Yogi A Neto Filho Mdos A Bendhack LM Touyz RM Tirapelli CR Ethanol induces vascular relaxation via redox-sensitive and nitric oxide-dependent pathways Vascul Pharmacol 2012 56 1-2 74 83 22155162 10.1016/j.vph.2011.11.006

[B28] Kvietys PR Granger DN Role of reactive oxygen and nitrogen species in the vascular responses to inflammation Free Radic Biol Med 2012 52 3 556 592 22154653 10.1016/j.freeradbiomed.2011.11.002 PMC3348846

